# The Ripple Effect: A Literature Review on Vicarious Trauma in Psychiatry Trainees

**DOI:** 10.1192/bjo.2025.10119

**Published:** 2025-06-20

**Authors:** Nafisa Darod

**Affiliations:** Greater Manchester Mental Health NHS Foundation Trust, Manchester, United Kingdom

## Abstract

**Aims:** Vicarious trauma (VT) is a significant concern among psychiatry trainees due to their frequent exposure to patients’ traumatic experiences. This exposure can lead to psychological distress, including symptoms resembling post-traumatic stress disorder (PTSD), emotional detachment, and burnout. This review explores the prevalence and impact of VT among psychiatry trainees and evaluates the effectiveness of support mechanisms such as supervision, peer support, and resilience-building strategies. By highlighting risk and protective factors, this paper informs psychiatry training programmes on safeguarding trainees’ well-being.

**Methods:** A literature review was conducted using databases including PubMed, PsycINFO, CINAHL, and Scopus. The search focused on peer-reviewed studies examining VT in mental health professionals, particularly psychiatry trainees. Key search terms included “vicarious trauma”, “psychiatry trainees”, “compassion fatigue”, and “support strategies”. A total of 39 studies were analysed, covering empirical research, qualitative investigations, and theoretical discussions.

**Results:** Findings indicate VT is widespread among psychiatry trainees, with many experiencing emotional distress, anxiety, and reduced professional efficacy. Risk factors include frequent exposure to trauma, high caseloads, inadequate supervision, and personal trauma history. VT impacts not only mental health but also professional relationships and career sustainability, with some trainees experiencing emotional exhaustion and compassion fatigue.

Several strategies help mitigate VT. Regular supervision provides a structured space for processing experiences, while peer support programmes reduce isolation and encourage shared coping mechanisms. Resilience-building strategies, such as mindfulness, self-care training, and structured debriefing, help trainees manage emotional challenges. Some experience vicarious resilience, where their work enhances professional fulfilment and emotional strength.

While research supports these interventions, methodological limitations – such as reliance on self-reported measures and cross-sectional designs – restrict definitive conclusions on long-term effectiveness. More robust, longitudinal studies are needed to assess sustained impacts.

**Conclusion:** Vicarious trauma significantly affects psychiatry trainees’ well-being, professional development, and retention. However, structured support – through supervision, peer networks, and resilience training – can mitigate its effects. Future research should assess the long-term effectiveness of interventions and explore how training institutions can better integrate trauma-informed approaches into curricula. By prioritising trainee well-being, psychiatry programmes can promote a more resilient mental health workforce.

